# Calibration of Linear Time-Varying Frequency Errors for Distributed ISAR Imaging Based on the Entropy Minimization Principle

**DOI:** 10.3390/s19061323

**Published:** 2019-03-16

**Authors:** Hailong Kang, Jun Li, Hongyan Zhao, Zhiyu Bao, Zehua Yu

**Affiliations:** 1 National Laboratory of Radar Signal Processing, Xidian University, Xi’an 710071, China; khlwork@163.com (H.K.); 15029035001@139.com (Z.B.); zehua_yu@163.com (Z.Y.); 2 College of Information and Computer, Taiyuan University of Technology, Taiyuan 030024, China; zhaohongyan@tyut.edu.cn

**Keywords:** distributed ISAR, entropy minimization principle, linear time-varying frequency errors, frequency error calibration

## Abstract

The inevitable frequency errors owing to the frequency mismatch of a transmitter and receiver oscillators could seriously deteriorate the imaging performance in distributed inverse synthetic aperture radar (ISAR) system. In this paper, for this issue, a novel method is proposed to calibrate the linear time-varying frequency errors (LTFE) between the transmitting node and the receiving node. The cost function is constructed based on the entropy minimization principle and the problem of LTFE calibration is transformed into cost function optimization. The frequency error coefficient, which minimizes the image entropy, is obtained by searching optimum solution in the solution space of cost function. Then, the original signal is calibrated by the frequency error coefficient. Finally, the effectiveness of the proposed method is demonstrated by simulation and real-data experiments.

## 1. Introduction

Inverse synthetic aperture radar (ISAR) is a type of all-weather, all-day, remote sensing radar [1,2] that plays a very important role in target imaging and recognition [3]. Multiple-input Multiple-output (MIMO) radar with widely separated antennas can be considered as a type of multistatic radar [4,5]. It is possible for this system to obtain spatial diversity of the targets’ radar cross section. Therefore, many scholars have studied multistatic ISAR and distributed MIMO-ISAR [6,7,8,9,10,11,12]. Compared with the traditional monostatic ISAR, distributed ISAR can achieve higher resolution and overcome some inherent drawbacks of monostatic ISAR by utilizing both spatial and temporal degrees of freedom. However, the frequency errors caused by frequency mismatch of the transmitter and the receiver oscillators become an important factor that restricts the application of distributed ISAR.

At present, the research studies on distributed ISAR are generally based on ideal conditions where the frequency errors between transmitter and receiver are not considered. The phase asynchronism caused by frequency errors in distributed radar system seriously degrades the performance of location [13], detection [14], and imaging. Literature, in the field of radar imaging, has studied the effect of frequency errors on multistatic SAR and MIMO radar imaging [15,16]. The first-order phase errors caused by the constant frequency errors only cause the image to shift in the cross-range direction without damaging the image quality. However, the quadratic phase errors caused by the linear time-varying frequency errors (LTFE) result in image defocus. Therefore, the calibration of the LTFE is mainly researched in this paper. In bistatic or multistatic SAR, phase synchronization is often achieved by designing special phase synchronization links to calibrate frequency errors, such as the direct-path echo method [17] and the pulse alternation method [18]. However, additional equipment is required to implement the frequency error calibration by these methods, which increases the complexity of the radar system. Few papers can be found in previous literature, in the field of frequency error calibration of distributed ISAR imaging.

In this paper, a method based on the entropy minimization principle is proposed to calibrate the LTFE. The image entropy is an important measure of image focusing performance. When the image entropy is smaller, the image focusing performance is better [19]. First, on the basis of the entropy minimization principle, the frequency error coefficient can be estimated by searching optimum solution in the solution space of cost function. Then, the original signal is calibrated by the estimated value of the frequency error coefficient. In the proposed method only radar echo is required and the additional hardware for the radar system is not required.

The remainder of this paper is organized as follows: The signal model of distributed ISAR and the imaging geometry are presented in Section 2. The basic idea of the proposed LTFE calibration method is explained in Section 3. The effectiveness of the proposed method will be demonstrated by simulation and real-data experiments in Section 4. Finally, our conclusions for this paper are summarized in Section 5.

## 2. Distributed ISAR System and Signal Model

The geometry of the distributed ISAR system and the signal model are introduced in this section. For simplicity and generality, two radars are used to construct the distributed ISAR systems. This model can be extended to the case of multiple radars. 

### 2.1. The Geometry of Distributed ISAR System

**Figure 1:**
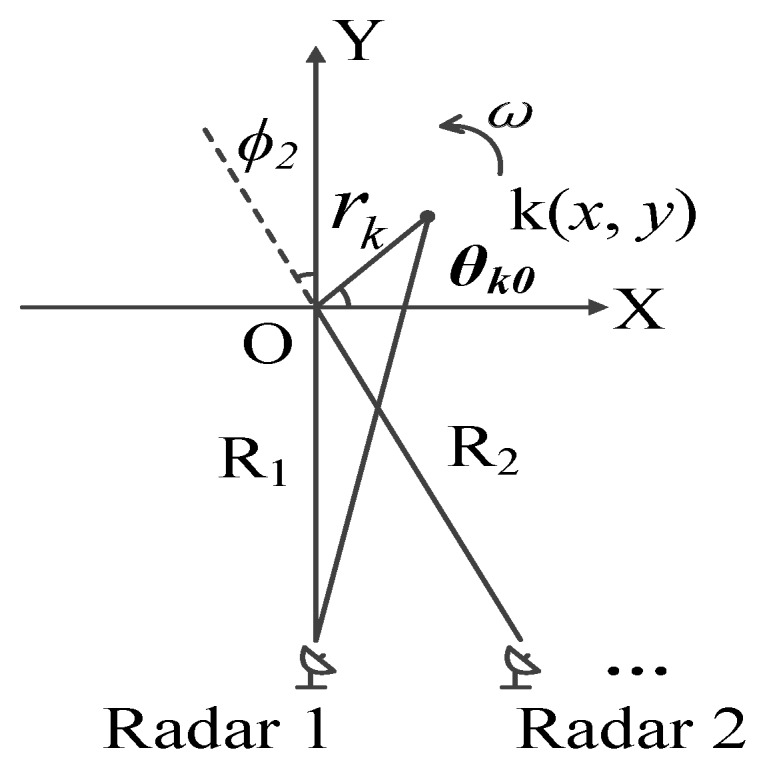
Geometry of distributed inverse synthetic aperture radar (ISAR).

The geometry of distributed ISAR system is shown in Figure 1. Radar 1 can transmit signals and receive signals, whereas radar 2 only receives signals. The O and k represent the reference center and generic scatterer on the target, respectively. The whole target is made up of K scatterers. The distance between the *k*th scatterer and the reference center O is $r_{k}$, the initial angle between the *k*th scatterer and the X axis is $\theta_{k0}$. The initial distance between radar 1, radar 2, and the reference center O is $R_{1}$ and $R_{2}$, respectively. $\phi_{n}$ denotes the angle between the line of sight (LOS) of the *n*th radar and Y axis. It is equivalent to a monostatic radar in that radar 1 acts as transmitter and radar 2 as receiver. It is assumed that the moving target has been transformed into a turntable model after motion compensation and the target rotates around the reference center with constant angular speed of ω for both radars.

### 2.2. Distributed ISAR Signal Model with LTFE

According to the geometry of distributed ISAR, the sum of the distance from the *i*th radar and the *j*th radar to the *k*th scatterer can be expressed as:

(1) $$R_{ijk}(t)=2\;\;\times \;\;(R_{ij}+r_{k}\sin(\theta_{k0}-\alpha_{ij}+\omega t)\cos\beta_{ij})$$

where $R_{ij}=\frac{1}{2}(R_{i}+R_{j})$ $R_{i}$ denotes the distance between the *i*th radar and the reference center O.

(2) $$\alpha_{ij}=(\phi_{i}+\phi_{j})/2$$

(3) $$\beta_{ij}=(\phi_{i}-\phi_{j})/2$$

The *i*th radar transmitter and the *j*th radar receiver can be equivalent to a monostatic radar, namely, the *n*th radar. So we can let $\alpha_{ij}=\alpha_{n}$, $\beta_{ij}\;=\;\;\beta_{n}$, $R_{ij}=R_{n}$. Then, the signal received by the *n*th radar (*n* = 0, …, N − 1) from the *k*th scatterer at time *t* is written as:

(4) $$\begin{array}{cc} s_{n}(t) & =\exp(-j\frac{2\pi }{\lambda }R_{ijk})\\ & =\exp(-j\frac{4\pi }{\lambda }\left[R_{n}+r_{k}\sin(\theta_{k0}-\alpha_{n}+\omega t)\cos\beta_{n}\right]{)\mathrm{rect}}_{T}(t) \end{array}$$

where ${\mathrm{rect}}_{T}(t)=\left\{ \begin{array}{l} 1\;\left|t\right|\leq \frac{1}{2}T\\ 0\;\left|t\right|>\frac{1}{2}T \end{array}\right.$ [5]. T is the total ISAR observation time for imaging. The rotation angle of target observed by the *n*th equivalent radar can be expressed as:

(5) $$\begin{array}{cc} \Delta \theta & =\theta_{k}^{n}(T/2)-\theta_{k}^{n}(-T/2)\\ & =(\theta_{k0}-\alpha_{n}+\omega T/2)-(\theta_{k0}-\alpha_{n}-\omega T/2)\\ & =\omega T \end{array}$$

where $\theta_{k}^{n}(T/2)$ represents the rotation angle of the *k*th scatterer at T/2 observed by the *n*th equivalent radar and $\theta_{k}^{n}(-T/2)$ is the rotation angle of the *k*th scatterer at −T/2 observed by the *n*th equivalent radar. When the rotation angle Δθ is small, Equation (4) can be approximately expressed as:

(6) $$s_{n}(t)≅\exp(-j\frac{4\pi }{\lambda }\left[R_{n}+(x_{r}^{k(n)}+x_{cr}^{k(n)}\omega t)\cos\beta_{n}\right]{)\mathrm{rect}}_{T}(t)$$

where $x_{r}^{k(n)}=r_{k}\sin(\theta_{k0}-\alpha_{n})$ and $x_{cr}^{k(n)}=r_{k}\cos(\theta_{k0}-\alpha_{n})$ are range and cross-range position on the imaging projection plane of the *n*th equivalent radar, respectively. In Equations (4) and (6), it is assumed that the local oscillators of both receiver and transmitter are perfect without frequency errors. In the actual distributed ISAR system, the carrier frequencies of transmitter and receiver are not always equal when the *i*th radar transmits and the *j*th radar receives, so the phase errors will occur after mixing. Assuming that the actual carrier frequency of the *i*th radar transmitter is $f_{ci}$ and that of the *j*th radar receiver is $f_{cj}$, the received signal of the *n*th equivalent radar after mixing can be rewritten as:

(7) $$s_{n1}(t)≅\exp(-j\frac{4\pi }{\lambda }\left[R_{n}+(x_{r}^{k(n)}+x_{cr}^{k(n)}\omega t)\cos\beta_{n}\right])\;\cdot \exp(j2\pi ((f_{ci}-f_{cj})t){)\mathrm{rect}}_{T}(t)$$

The signal model with frequency errors is expressed in Equation (7). In this paper, we focus on the LTFE which can be defined as follow:

(8) $$\delta f=f_{ci}-f_{cj}=f_{0}(1+\rho_{i}t)-f_{0}(1-\rho_{j}t)=f_{0}\rho_{i}t+f_{0}\rho_{j}t=(A_{i}+A_{j})t$$

where $\rho_{i}$ and $\rho_{j}$ are stability of the *i*th transmitter oscillator and the *j*th receiver oscillator, respectively. In Equation (8), $A_{i}$ equals $f_{0}\rho_{i}$ and $A_{j}$ equals $f_{0}\rho_{j}$. $f_{0}$ is the ideal carrier frequency of transmitter and receiver. After substituting Equation (8) into the second exponential term of Equation (7), we can get the following result:

(9) $$\exp(j2\pi (f_{ci}-f_{cj})t)=\exp(j2\pi (A_{i}+A_{j})t^{2})$$

The quadratic phase term in Equation (9) is produced by the LTFE which is the main reason for the image defocusing. Therefore, the focus of this paper is to eliminate the influence of quadratic phase terms on distributed ISAR imaging by LTFE calibration. After substituting Equation (9) into Equation (7), the echo signal model with LTFE can be re-expressed as:

(10) $$s_{n1}(t)≅\exp(-j\frac{4\pi }{\lambda }\left[R_{n}+(x_{r}^{k(n)}+x_{cr}^{k(n)}\omega t)\cos\beta_{n}\right])\;\cdot \exp(j2\pi ((A_{i}+A_{j})t^{2}){)\mathrm{rect}}_{T}(t)$$

According to Equation (6), it is known that there is a Fourier transform relationship between the cross-range coordinate and time t. The cross-range focusing can be achieved by Fourier transform. The target rotation angles of each radar during T are ωT. These rotation angles are not completely overlapping as different radars have different observation angles. Therefore, a larger aperture can be synthesized by rearranging and adding echo data of different radars. According to the fusion scheme in literature [5], the fusion signal can be expressed as:

(11) $$s_{comb}(t)=\sum_{n=1}^{N}\exp(j\frac{4\pi }{\lambda }R_{n})\cdot s_{n}(t-t_{n}){\mathrm{rect}}_{T_{n}}(t-t_{n}+\Delta t_{n})$$

where $t_{n}=(\alpha_{1}+\alpha_{N})/2\omega -\alpha_{n}/\omega$, $T_{n}=(\alpha_{n-1}-\alpha_{n+1})/2\omega$ and $\Delta t_{n}=(\alpha_{n-1}+\alpha_{n+1})/4\omega -\alpha_{n}/2\omega$, with $\alpha_{0}=\alpha_{1}+\omega T$ and $\alpha_{N+1}=\alpha_{N}-\omega T$. After processing the echo signal of each radar according to Equation (11), the global fused signal can be re-expressed as Equation (12):

(12) $$\begin{array}{cc} s_{comb}(t) & =\sum_{n=1}^{N}\exp(-j\frac{4\pi }{\lambda }\left[r_{k}\sin(\theta_{k0}+\omega t-\frac{\alpha_{1}+\alpha_{N}}{2})\cos\beta_{n}\right])\cdot {\mathrm{rect}}_{T}(t-t_{n}){\mathrm{rect}}_{T_{n}}(t-t_{n}+\Delta t_{n})\\ & =\exp(-\frac{4\pi }{\lambda }\left[r_{k}\sin(\theta_{k0}+\omega t-\frac{\alpha_{1}+\alpha_{N}}{2})\cos\beta_{0}\right])\cdot \sum_{n=1}^{N}{\mathrm{rect}}_{T}(t-t_{n}){\mathrm{rect}}_{T_{n}}(t-t_{n}+\Delta t_{n})\\ & =\exp(-\frac{4\pi }{\lambda }\left[r_{k}\sin(\theta_{k0}+\omega t-\frac{\alpha_{1}+\alpha_{N}}{2})\cos\beta_{0}\right])\cdot {\mathrm{rect}}_{T+(\alpha_{1}-\alpha_{N})/\omega }(t) \end{array}$$

$\beta_{0}$, $\beta_{n}$ and $\beta_{n-1}$ are assumed to be equal constants in Equation (12). In order to avoid gaps in the overall view angle, it is required that $\alpha_{n-1}-\alpha_{n}\leq \;\omega T$ in Equations (11) and (12). According to Equation (12), it can be observed that the fusion result of multiple radar signals is equivalent to increasing the observation time of single radar. When there are LTFE, the fusion result of multiple radar signals is:

(13) $$s_{comb1}(t)=\sum_{n=1}^{N}\exp(j\frac{4\pi }{\lambda }R_{n})\cdot s_{n1}(t-t_{n}){\mathrm{rect}}_{T_{n}}(t-t_{n}+\Delta t_{n})$$

Comparing Equation (13) with Equation (11), the signal fusion in the presence of LTFE is equivalent to adding a quadratic phase term to the echo of each radar before signal fusion. If the quadratic phase term of the *n*th equivalent radar echo is not removed before signal fusion, it will still exist in the fusion signal, which will degrade the performance of fuse signal imaging. Therefore, the frequency errors of each equivalent radar echo signal should be calibrated before signal fusion.

## 3. Calibration Method of LTFE

In this section, the LTFE will be calibrated based on the entropy minimization principle. Here, H shown in Equation (14) is the frequency error calibration term:

(14) $$H=\exp(-j2\pi \Delta At^{2})$$

where ΔA is the frequency error coefficient. The expression of signal with LTFE in Equation (10) multiplied by Equation (14) is:

(15) $$\begin{array}{c} s_{n1compen}(t)=s_{n1}(t)\times H≅\exp(-j\frac{4\pi }{\lambda }\left[R_{n}+(x_{r}^{k(n)}+x_{cr}^{k(n)}\omega t)\cos\beta_{n}\right])\\ \cdot \exp(j2\pi ((A_{i}+A_{j})-\Delta A)t^{2}{)\mathrm{rect}}_{T}(t) \end{array}$$

According to Equation (15), it is obvious that the LTFE is completely calibrated when $(A_{i}+A_{j})=\Delta A$, namely, quadratic phase coefficient is zero. Therefore, the key to this problem is to find an accurate ΔA to calibrate the original signal before signal fusion and imaging.

**Figure 2:**
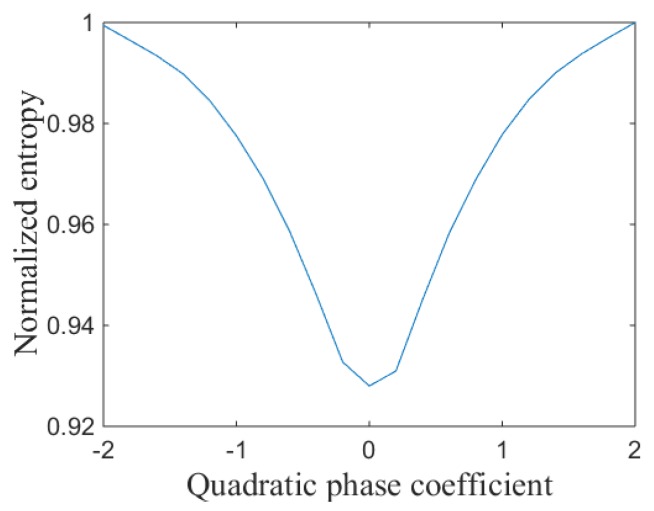
Relationship between normalized entropy and quadratic phase coefficient.

The quadratic phase errors produced by the LTFE can defocus the image, so the image entropy will increase. The image focusing performance is the best when the quadratic phase coefficient $\left((A_{i}+A_{j})-\Delta A\right)$ is zero. In such a case, the image entropy also reaches minimum. The relationship between image entropy and quadratic phase coefficient is shown in Figure 2.

Figure 2 shows that the image entropy reaches minimum when the LTFE is calibrated by ΔA. 

Therefore, the entropy minimization principle can be employed to estimate ΔA. For estimating ΔA, the image $I_{0}$ shown in Equation (16) needs to be obtained by Fourier transform:

(16) $$I_{0}=\mathrm{FT}(s_{n1compen}(t))$$

where FT (·) represents Fourier transform operation. Then, the entropy function is expressed as:

(17) $$\mathrm{Entropy}(\Delta A)=-\sum_{m=1}^{M}\sum_{n=1}^{N}I(m,n)\ln[I(m,n)]$$

where $I(m,n)=\left|I_{0}(m,n)\right|/\sum_{m=1}^{M}\sum_{n=1}^{N}\left|I_{0}(m,n)\right|$. $I_{0}(m,n)$ is the gray value of the (m,n) point on the image $I_{0}$. Image size is M×N. The LTFE calibration method proposed in this paper is to estimate the the frequency error coefficient by minimizing the image entropy. Then the estimated value of the frequency error coefficient is used to calibrate the LTFE of the original signal. Therefore, the entropy function can be used as the cost function. The problem of LTFE calibration can be treated as the problem of cost function optimization. The cost function is expressed as:

(18) $$\Delta \hat{A}=\arg\underset{\Delta A}{\min}\{\mathrm{Entropy}(\Delta A)\}$$

The frequency error coefficient ΔA can be obtained by searching the optimum solution in the solution space of the cost function in Equation (18). After calibrating the LTFE with the estimated value $\Delta \hat{A}$, the signals are fused for further imaging. The imaging result will have a good focus performance.

The main steps of the proposed method are summarized as follows:

**Step 1**  set the initial value, search scope and search step size of ΔA.
**Step 2**  according to Equation (14) and (15), $\Delta A_{m}$ in every search step is used to compensate the original signal ($\Delta A_{m}$ is the *m*th possible estimate value of ΔA).
**Step 3**  image with compensated signal and calculate image entropy $E_{m}$.
**Step 4**  complete the search in the whole search range of ΔA, compare the image entropy of all the imaging results, and select the $\Delta A_{m}$ corresponding to the minimum entropy as the estimated value of ΔA.
**Step 5**  calibrate the signal $s_{n1}(\hat{t},t_{m})$ of the *n*th radar with the estimated value of ΔA obtained in step 4.
**Step 6**  calibrate the echo signal of every equivalent radar, and fuse the signals for imaging finally according to the method of step 1–5.

**Figure 3:**
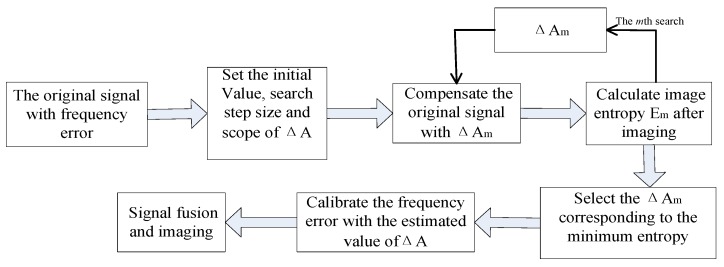
Flow chart of calibration method for frequency error based on entropy minimization principle.

The flow chart which illustrates the procedure of the proposed method is shown in Figure 3.

## 4. Numerical Simulation and Analysis

In this section, the effectiveness of the proposed method is demonstrated by simulation and real-data experiments. In the simulation, the simplest distributed ISAR system is used as shown in Figure 1. Radar 1 is a self-transmitting and self-receiving radar which has no frequency errors. Radar 2 receives signals transmitted by radar 1, therefore in this case, the LTFE caused by the frequency mismatch between the transmitter and the receiver oscillators must be calibrated. Then, the received signals of radar 1 and radar 2 are fused.

### 4.1. Simulation 1: The Effectiveness of the Proposed Method

Simulation parameters are given in Table 1.

**Figure 4:**
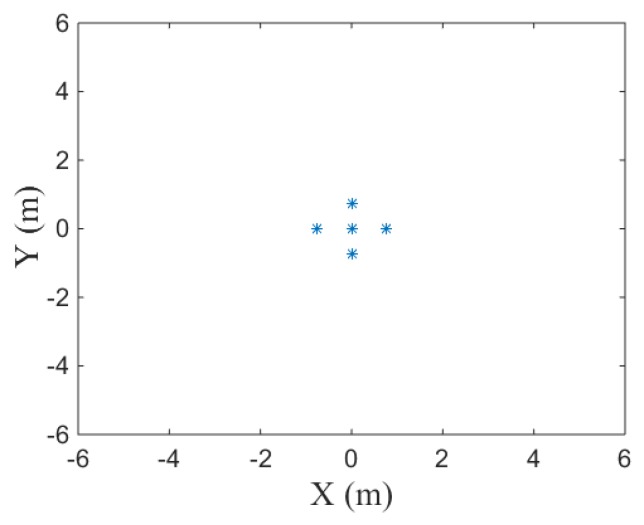
Target model.

**Figure 5:**
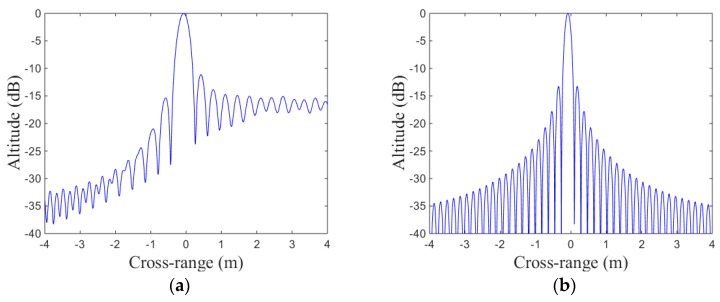
Cross-range point spread function (PSF): (**a**) cross-range PSF with LTFE; (**b**) cross-range PSF after calibrating LTFE.

**Figure 6:**
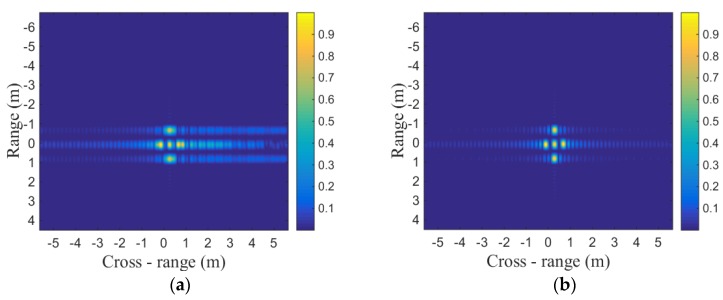
Two-dimensional imaging results: (**a**) imaging results of fused signal with LTFE; (**b**) imaging results of fused signal after calibrating frequency error.

The target model used in the simulation is shown in Figure 4. In Figure 5, cross-range point spread function (PSF) with LTFE is compared with the calibrated one. It is demonstrated that the LTFE seriously deteriorate the spectrum of cross-range and the LTFE can be calibrated effectively by the proposed method. The performance of cross-range PSF is improved when the LTFE is calibrated by the proposed method. According to the two-dimensional imaging results shown in Figure 6, it can be seen that the focusing performance of Figure 6b is much better than that of Figure 6a. 

Image entropy and image contrast [20] are two objective metrics used to evaluate image quality. In order to quantitatively prove the effectiveness of the proposed method from a mathematical point of view, we employ image entropy and image contrast to further explain the simulation results. The image entropy is defined in Equation (17). The image contrast is defined as follows:

(19) $$\mathrm{contrast}=\frac{\sqrt{E\left\{{\left(\left|u_{mn}\right|-E\left\{\left|u_{mn}\right|\right\}\right)}^{2}\right\}}}{E\left\{\left|u_{mn}\right|\right\}}$$

where $u_{mn}$ is the amplitude of each pixel and E{⋅} represents the mean operation. According to Equations (17) and (19), the image entropy and image contrast shown in Figure 5 are listed in Table 2 and Table 3, respectively.

Table 2 shows that the image entropy shown in Figure 5b is smaller than that shown in Figure 5a, in other words, the focusing performance shown in Figure 5b is better than that shown in Figure 5a.

The bigger the image contrast is, the better the image focusing performance is. Table 3 shows that the image entropy shown in Figure 5b is bigger than that shown in Figure 5a, which means that shown in Figure 5b has better focusing performance than that shown in Figure 5a. Both image entropy and image contrast prove that the focusing performance shown in Figure 5b is better than that shown in Figure 5a, namely, the LTFE can be calibrated effectively by the proposed method.

Similar to Figure 5, the image entropy and image contrast shown in Figure 6 are listed in Table 4 and Table 5, respectively.

As listed in Table 4 and Table 5, the image entropy shown in Figure 6b is smaller than that shown in Figure 6a, and the image contrast shown in Figure 6b is larger than that shown in Figure 6a. In other words, the focusing performance shown in Figure 6b is better. These simulation results demonstrate the effectiveness of the proposed method.

### 4.2. Simulation 2: Demonstration by Real-Data

The target model used in the simulation 1 is a scatterer model, which is not completely consistent with the true situation. Accordingly, Yake-42 real-data [21,22] is utilized to further demonstrate the effectiveness of the proposed method. The Yake-42 aircraft is 36.8 m in length and 34.88 m in width. It flies smoothly along a straight line at an altitude of 4 km from the ground. A ground-based imaging radar is used to acquire the echo data of the Yake-42 aircraft. Some important radar parameters of the Yake-42 real-data are listed in Table 6.

The Yake-42 real-data is a single-sensor data, so it cannot be directly used in distributed ISAR fusion imaging. We need to emulate the two sensors’ distributed ISAR acquisition with the Yake-42 real-data. To accomplish this, the Yake-42 real-data of 256 continuous pulses is divided into two parts. The data of one to 128 pulses is used as radar 1 echo data and the data of 129 to 256 pulses is used as radar 2 echo data. The Yake-42 data of 256 continuous pulses is equivalent to the echo data received simultaneously by two sensors during 1.28 s. In the true distributed ISAR system, echo data of radar 2 has frequency errors. However, Yake-42 data is received by a self-transmitting and self-receiving radar with no frequency errors. Therefore, it is necessary to add a LTFE term to the equivalent echo data of radar 2 and set the frequency error coefficient as 5 Hz/s^2^. The echo data of two sensors’ distributed ISAR system will be emulated based on the aforementioned processing. Then, the distributed ISAR real-data can be used to demonstrate the effectiveness of the proposed method. 

**Figure 7:**
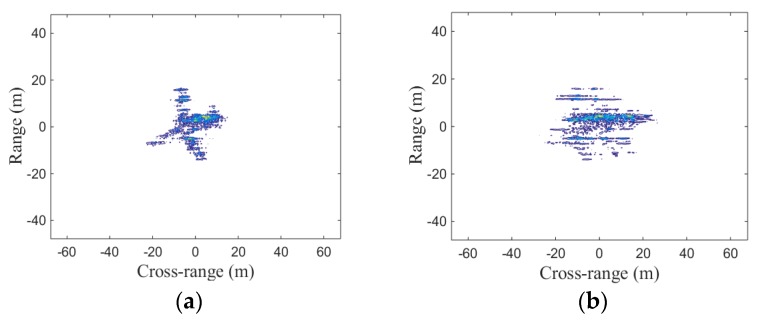

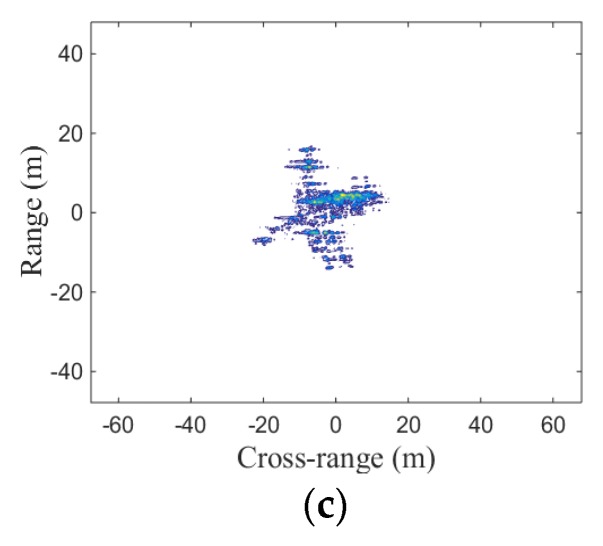
Coherent fusion imaging results of real-data: (**a**) imaging result of real-data without LTFE; (**b**) imaging result of real-data with LTFE; (**c**) imaging result of real-data after calibrating LTFE.

Figure 7a shows the imaging result of distributed ISAR real-data without LTFE. It is obvious that the image has a good focusing performance. In practice, frequency errors inevitably exist in the distributed ISAR system because of the frequency mismatch between the transmitter and the receiver oscillators. Figure 7b shows the imaging result of distributed ISAR real-data with LTFE. It can be seen that the LTFE cause the image defocus and the imaging performance is seriously deteriorated. The imaging result of distributed ISAR real-data after calibrating the LTFE by the proposed method is shown in Figure 7c. The focusing performance shown in Figure 7c is better than that shown in Figure 7b, which demonstrates that the proposed method can effectively calibrate the LTFE and improve the imaging performance. It can be seen from the comparison of the imaging results of Figure 7a with Figure 7c, the proposed method is effective for the real-data. For further verification, the image entropy and image contrast are listed in Table 7 and Table 8, respectively.

The data in Table 7 and Table 8, show that the image entropy shown in Figure 7c is smaller than that shown in Figure 7b, while the image contrast shown in Figure 7c is bigger than that shown in Figure 7b. These two metrics demonstrate that the focusing performance shown in Figure 7c is better than that shown in Figure 7b and the proposed method in this paper can effectively calibrate the LTFE.

**Figure 8:**
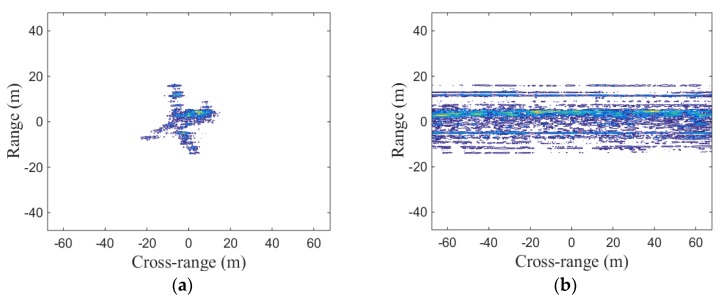

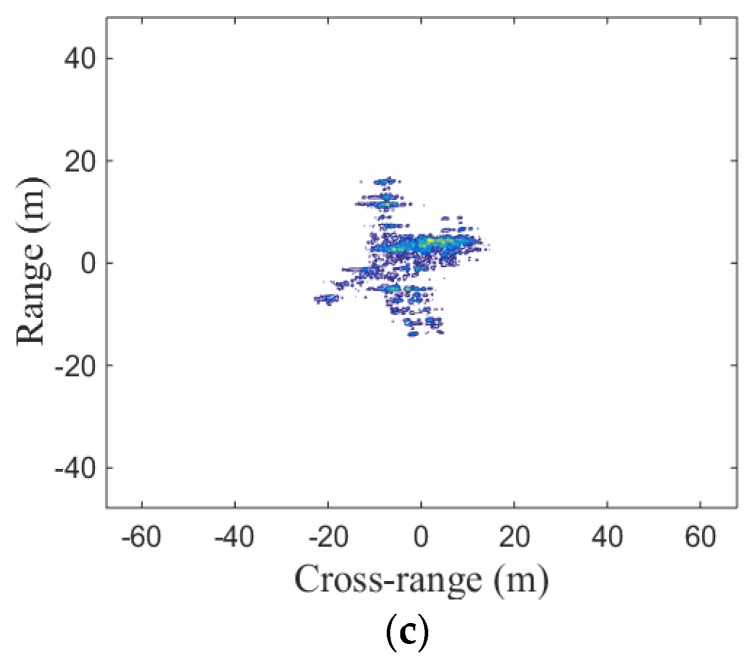
Coherent fusion imaging results under large LTFE: (**a**) imaging result without large LTFE; (**b**) imaging result with large LTFE; (**c**) imaging result after calibrating large LTFE.

In order to prove that the proposed method can effectively calibrate the LTFE in different sizes, another experiment under the condition of large LTFE is shown in Figure 8. In this experiment, the frequency error coefficient is set as 15 Hz/s^2^.

Figure 8b shows that the image is completely out of focus when there is a large LTFE. As shown in Figure 8c, a well-focused image is obtained after calibrating the large LTFE by the method proposed in this paper. The image entropy and image contrast of Figure 8 are listed in Table 9 and Table 10, respectively.

The image entropy listed in Table 9 and the image contrast listed in Table 10 also prove that the focusing performance shown in Figure 8c is better than the focusing performance shown in Figure 8b, namely, the proposed method in this paper can still effectively calibrate LTFE and improve image performance under the condition of large LTFE.

## 5. Conclusions

Frequency errors in distributed ISAR systems degrade the image performance. For this issue in this paper, an error calibration method based on entropy minimization principle is proposed that can calibrate the LTFE of distributed ISAR system and realize phase synchronization. The distributed ISAR fusion imaging performance is improved significantly after calibrating the LTFE by the proposed method. Only radar echo is required in the proposed method so the additional hardware for the radar system is not required. The numerical simulation results show that the LTFE in the distributed ISAR can be calibrated effectively by the proposed method.

## Figures and Tables

**Table 1:** Simulation parameters.

Carrier frequency *f*_0_	10 GHz
Bandwidth B	400 MHz
Acquisition time T	1.59 s
Rotational speed *ω*	0.36 °/s
Pulse width T_p_	0.64 μs
Stability of oscillator	$\rho_{1}\;=\;10^{-10},\;\rho_{2}\;=\;10^{-10}$
Error coefficient Δ*A*	2 Hz/s^2^

**Table 2:** Image entropy of Figure 5.

Figure	Image Entropy
5a (with LTFE)	7.0870
5b (after calibrating)	6.3900

**Table 3:** Image contrast of Figure 5.

Figure	Image Contrast
5a (with LTFE)	1.2790
5b (after calibrating)	2.7660

**Table 4:** Image entropy of Figure 6.

Figure	Image Entropy
6a (with LTFE)	10.3580
6b (after calibrating)	7.043

**Table 5:** Image contrast of Figure 6.

Figure	Image Contrast
6a (with LTFE)	2.4950
6b (after calibrating)	3.4238

**Table 6:** Radar parameters of Yake-42 real-data.

Carrier frequency	5.5 GHz
Bandwidth	400 MHz
Pulse width	25.6 μs
Prf	100 Hz
Number of pulses	256

**Table 7:** Image entropy of Figure 7.

Figure	Image Entropy
7a (without LTFE)	11.4082
7b (with LTFE)	11.7183
7c (after calibrating)	11.4082

**Table 8:** Image contrast of Figure 7.

Figure	Image Contrast
7a (without LTFE)	2.1338
7b (with LTFE)	1.8582
7c (after calibrating)	2.1338

**Table 9:** Image entropy of Figure 8.

Figure	Image Entropy
8a (without LTFE)	11.4082
8b (with LTFE)	11.9232
8c (after calibrating)	11.4082

**Table 10:** Image contrast of Figure 8.

Figure	Image Contrast
8a (without LTFE)	2.1338
8b (with LTFE)	1.4074
8c (after calibrating)	2.1338
